# Downregulation of hsa_circTLK1 represses non-small cell lung cancer progression by regulating miR-876-3p/SRSF7 axis

**DOI:** 10.1016/j.heliyon.2024.e31972

**Published:** 2024-05-25

**Authors:** Xinzhe Dong, Hui Tian, Peng Ren, Yanxia Liu, Lin Wang

**Affiliations:** a Department of Radiation Oncology, Qilu Hospital of Shandong University, Cheeloo College of Medicine, Shandong University, China; b Department of Oncology, Shengli Oil Central Hospital, Dongying, China

**Keywords:** hsa_circTLK1, miR-876-3p, SRSF7, non-small cell lung cancer

## Abstract

**Background:**

This study clarified the expression of cicrTLK1 in non-small cell lung cancer (NSCLC) and explored its role in cancer growth, metastasis and immune escape, providing a potential molecular target and theoretical basis for NSCLC treatment.

**Methods:**

The expression levels of circTLK1, miR-876-3p and SRSF7 were determined by RT-qPCR assay. The localization of circTLK1 in NSCLC cells was determined by FISH assay. EdU and cell plate clone formation assay were applied to explore cell proliferation. Wound healing test and Transwell assay were applied to measure the migration and invasion ability. Cell apoptosis rate was detected by FCM assay. Western blotting assay was adopted to measure the protein expression of SRSF7. Dual-luciferase reporter gene assay was applied to assess the interaction between miR-876-3p and circTLK1, and between miR-876-3p and SRSF7. The ability of cirTLK1 to regulate tumor formation *in vivo* was examined by tumor transplantation experiments in nude mice.

**Results:**

The relative expression of circTLK1 was increased in NSCLC cell lines. Knockdown of circTLK1 prohibited the proliferation, migration, and invasion, and promoted apoptosis rate, but miR-876-3p inhibitor reversed the effects of circTLK1 knockdown. In addition, silencing of circTLK1 overtly restrained the growth of transplanted tumors in *vivo*, and inhibited immune escape. In addition, circTLK1 interacted with miR-876-3p, and SRSF7 was concluded to be the target gene of miR-876-3p.

**Conclusion:**

In this study, we researched the inhibitory effect of circTLK1knockdown on NSCLC progression and immune escape, and further elucidated the potential regulatory mechanism of circTLK1/miR876-3p/SRSF7 axis.

## 1 Introduction

Lung cancer, one of the most common cancers in clinical practice, is still on the rise in morbidity and mortality. It has become a serious disease threatening the health of all mankind. The latest data show that lung cancer is the main cause of death in male cancer patients, and the mortality rate of female lung cancer patients has also exceeded that of breast cancer [1,2]. In the past 30 years from 1990 to 2019, the incidence and mortality of lung cancer in China have been on the rise, ranking among the highest in the world [3]. China is estimated to have 870,982 new cases of lung cancer and 130,108 deaths from lung cancer in 2022 [4]. Non-small cell lung cancer (NSCLC) accounts for more than 85 percent of all diagnosed lung cancer cases. In the early stage of lung cancer, there are often no obvious clinical symptoms, and most cases only appear as lung nodules on CT images. Most patients with lung cancer are in the advanced stage of lung cancer (Ⅲ/Ⅳ stage) when diagnosed, and the prognosis is often poor, with a 5-year survival rate of only 19.7 % [5]. Therefore, looking for molecular markers related to the development of lung cancer is of great significance for early clinical intervention and improving the prognosis of patients with lung cancer.

CircRNA is widely found in mammalian cells and is a kind of ncRNA without a 5 'cap and a 3' polyA-tail structure. Unlike typical linear RNA, circRNA has a closed ring structure, which is formed by the end to end of covalent bonds, so it is not easy to be degraded by ribonuclease. The average half-life of circRNA in plasma exceeds 48 h, which is longer than that of mRNA [6]. There is evidence that the abnormal expression of circRNAs is closely related to the occurrence and development of lung cancer. Li et al. identified 43 up-regulated circRNAs and 78 down-regulated circRNAs in NSCLC [7]. Furthermore, has_circ_100395 suppresses the multiplication, migration, and invasion by the regulation of miR-1228/TCF21 [8]. However, the potential role of most circRNAs in the development of NSCLC remains unclear. Hsa_circTLK1 is derived from TLK1 mRNA, located on chromosome 2: 171879381-171902872. In renal cell carcinoma, the expression of circTLK1 is elevated and positively associated with poor prognosis and distant metastasis [9]. Furthermore, the inhibition effect of circTLK1 knockdown has also been reported in glioma progression [10]. Whether circTLK1 is involved in NSCLC has not been determined, therefore, this study aimed to explore the regulatory role of cirTLK1 on NSCLC cells.

The interaction of complementary sequences between the circRNA and miRNA leads to the adsorption of miRNA, thus preventing miRNA from binding to its target mRNAs and losing its ability to inhibit gene expression [11]. Recently, a large number of studies have reported the structure, function, biogenesis and mechanism of miRNAs, which can regulate protein expression at the post-transcriptional level [12]. MiR-876-3p has been reported to be involved in the progression of various cancers, including breast cancer [13], ovarian cancer [14], and colon cancer [15]. A previous study have confirmed the role of miR-876-3p in NSCLC [16], but whether circTLK1 regulates miR-876-3p remains unclear.

Therefore, in this study, we focused on the effect of circTLK1 on the progression of NSCLC cells. Moreover, the relationship between circTLK1 and miR-876-3p, as well as between miR-876-3p and SRSF7 was verified. This study aimed to explore the possible molecular mechanism of circTLK1 in NSCLC, in order to provide a new idea for the diagnosis and treatment of NSCLC.

## 2 Materials and methods

### 2.1 Cell lines and cell transfection

NSCLC cell lines (A549, SK-MES-1, NCI–H23, NCI–H1734) were purchased from the cell bank of Chinese Academy of Sciences. Normal bronchial epithelial cells HBE was purchased from EK-Bioscience (Shanghai, China). In the Roswell Park Memorial Institute (RPMI 1640 medium, Hyclone USA) containing 10 % fetal bovine serum (FBS). CircTLK1 overexpression plasmid was constructed by using the pcD-ciR plasmid as vector (GENESEED, China). Si-circTLK1, si-NC, miR-876-3p mimic, mimic NC, miR-876-3p inhibitor, inhibitor NC, si-SRSF7 and siRNA control were designed and synthesized by GenePharma (Shanghai, China). The cells were inoculated into 6-well plates and transfected with by liposome method (Lipofectamine TM 2000, Invitrogen, USA). The cells were collected after 48 h of culture for subsequent experiments.

### 2.2 RT-qPCR

Total RNA was extracted from NSCLC cell lines using Trizol reagent (Invitrogen, USA). The purity and concentration of the extracted RNA were measured by spectrophotometer (NanoDrop ND-100, USA). By using the Prime Script™ RT reagent Kit (TaKaRa, Japan), total RNA was reverse-transcribed. RT-qPCR was performed using QuantStudio Real-time PCR instrument with SYBR® Premix Ex Taq^TM^ Ⅱ reagent (TaKaRa, Japan). PCR reaction conditions: predenaturation at 95 °C for 15min; 95 °C for 10 s, 66°Cffor 32 s, a total of 45 cycles, extended at 72 °C for 30min. The expression levels of circTLK1, miR-876-3p and SRSF7 were calculated by 2^−ΔΔCT^. The primer sequences are listed in Table 1.

**Table 1:** Primer sequences used for RT-qPCR.

Primer name	Sequence (5’- 3’)
CircTLK1-Forward(F)	CAGTCAATGGAGCAGAGAA
CircTLK1-Reverse(R)	CCATTCTTGTTGCCTTTTTG
miR-876-3p-F	TGGTGGTTTACAAAGTAATTCA
miR-876-3p-R	AATTACTTTGTAAACCACCATT
SRSF7-F	GACGAAGGTCAAGGTCAGCA
SRSF7-R	CCTCGACGGGGATTGGAAAT
U6–F	CTCGCTTCGGCAGCACA
U6-R	AACGCTTCACGAATTTGCG
β-actin-F	CTTCGCGGGCGACGAT
β-actin-R	CCACATAGGAATCCTTCTGACC

### 2.3 RNase R

The extracted RNA was incubated with RNase R (GSPure® RNase R, GENESEED, China) at 37 °C for 0–30min and at 70 °C for 10min to inactivate the enzyme. The mRNA expression of circTLK1 and its host gene TLK1 was detected by RT-qPCR.

### 2.4 Fluorescence in situ hybridization (FISH)

The 1 × 10^4^ cells were inoculated in 24-well plates and cultured overnight in a cell incubator. 100 μL 4 % paraformaldehyde and 100 μL 0.5 % TritonX-100 were added to each well at room temperature. Fluor 488 labeled hsa_circTLK1 probe and Alexa Fluor 555 labeled miR-876-3p probe were synthesized by Gene-Pharma. 100 μL DAPI solution (Beyotime, China) was added to stain away from light for 5min. The images were observed and photographed under a fluorescence (LEICA, Germany).

### 2.5 Nucleo-plasmic partitioning

Cell suspension of each group was centrifuged at 1000 rpm for 5min, and the supernatant was removed. RNA was extracted from the nucleus and cytoplasm according to the nucleoplasmic extraction kit (Beyotime, China).

### 2.6 5-Ethynyl-2’-deoxyuridine (EdU)

During the logarithmic growth phase, cells were inoculated in 96-well plates with 1 × 10^5^ cells per well overnight. According to the EDU-555 test kit, cells were incubated with 50 mM EdU at 37 °C for 2h. The cells were fixed with 4 % paraformaldehyde and then stained with Hoechst33342 reagent and Apollo. Five fields were randomly selected under a fluorescence microscope (Olymous, Japan) to calculate the percentage of EdU labeled cells.

### 2.7 Transwell assay

The cell density of each group was adjusted to 5 × 10^4^ cells/mL. The Transwell chamber was placed on a 24-well plate. 100 μL cell suspension was added to the upper chamber coated with Matrigel, and the lower chamber was added with 500 μL medium. After 24h of culture, paraformaldehyde (Solarbio, China) was added to fix for 25 min and crystal violet (Sigama, USA) was used to stain for 15 min. Cells were counted in 5 random fields and photographed under an inverted microscope (Olympus, Japan).

### 2.8 Cell plate clone formation assay

The cells in each group were digested with trypsin 48h after transfection, centrifuged at 1000 rpm for 5min at 4 °C. The supernatant was discarded. The cells were resuspended in complete culture medium after discarding the supernatant. The cells were inoculated on 6-well plates with 500 cells per well and cultured in a 5 % CO_2_ cell incubator at 37 °C for 2–3 weeks. When visible clones appeared, the culture was terminated. After washing with PBS buffer for 1-2 times, formaldehyde was added to the fixation for 15 min. 2 mL crystal violet (2 %) was added to each well for 2 h, then washed off the staining solution with clean water. After dried naturally at room temperature, the clones were photographed (Nikon) and counted using ImageJ (USA).

### 2.9 Wound healing test

An appropriate amount of cells (5 × 10^5^) were laid in the six-well plate for overnight growth. The next day, the medium was changed into serum-free medium. After 12 h of starvation, a cross was drawn in the center with the tip of a 200 μL gun. The culture was carried out in 5 % CO_2_ and 37 °C incubator. Photos were taken at 0 and 24 h under the microscope (Olympus, Japan), respectively.

### 2.10 FCM

After 48 h of transfection, 1 × 10^6^ resuspended cells were rinsed twice with PBS pre-cooled at 4 °C. Then 5 μL AnnexinV-FITC solution (KeyGEN BioTECH, China) and 5 μL PI solution (4 μg/mL, KeyGEN BioTECH, China) were added. The cells were incubated in dark at room temperature for 30min, and then the apoptosis rate was detected by flow cytometry (FACS caliber, BD, USA).

### 2.11 Western blotting assay

The cells of each group were cultured for 48 h after transfection, centrifuged at 800 rpm for 5min, and the proteins were extracted by RIPA lysate. The protein concentration of each group was determined by BCA method. Then, SDS-PAGE electrophoresis was carried out. After transmembrane sealing, the primary antibody (anti-β-actin: ab179467, 1:5000, Abcam, UK; anti-SRSF7: ab138022, 1:1000, Abcam, UK) was sealed overnight at 4 °C, and the secondary antibody (ab6728, 1:2000, Abcam, UK) was sealed for 1 h. PVDF membrane was incubated with ECL luminescent solution, and then imaged on a luminous imager (Amersham Imager 600, GE, USA).

### 2.12 Dual-luciferase reporter assay

A549 cells and NCI–H1734 cells were inoculated into 24-well plates, and WT-circTLK1 and MUT-circTLK1 were co-transfected with miR-876-3p mimic or miR-NC using liposome method, respectively. After 24 h of culture, cells were collected and luciferase activity was detected according to the operation instructions of the double luciferase activity detection kit.

### 2.13 Isolation and culture of CD8^+^ T cells

CD8+T cells were isolated from whole blood of healthy individuals using the EasySep^TM^ kit (STEMCELL Technologies, Canada). CD8^+^ T cells were cultured with RPMI-1640 medium containing 10 % FBS before being used in further experiments.

### 2.14 Enzyme-linked immunosorbent assay (ELISA)

The expression levels of IFN-γ, IL-2 and TNF-α were detected by ELISA. si-NC and si-circTLK1 were transferred into A549 and NCI–H1734 cells according to the previous method, and then CD8^+^ T cells were added and co-cultured for 48 h. The co-cultured cells were collected and centrifuged at 15000 rpm/min for 3min to collect the supernatant. According to the kit instructions (Shanghai Enzyme-linked Biotechnology Co., Ltd, China), the standard curves of IFN-γ, IL-2 and TNF-α were drawn. The absorbance value was detected at 450 nm with an enzyme marker (Multiskan FC, Thermo Fisher, USA).

### 2.15 Tumor transplantation experiment

Sh-RNA-circTLK1 stable cell line was synthesized by Shanghai Jima Pharmaceutical Technology Co., LTD. The suspension cells (2 × 10^5^ cells/well) were inoculated in 6-well plates and placed in incubators overnight. 1 mL culture medium containing 5ug/mL Polybrene was added to each well, and then the culture was continued in the incubator for 48 h. After transfection, the stable cells were screened with 1ug/ml purine toxin.

The male nude mice used in this study were purchased from Vital River (China) and were 4–6 weeks old. They were routinely raised in SPF animal house of Animal center of our hospital. 200 μL cell suspension was injected into the underarm of nude mice. Tumor size was measured at 5-day intervals and tumor volume was calculated. 30 days after inoculation, the nude mice were euthanized, the tumors were removed, measured and photographed. All animals were treated in accordance with the Guidelines for the Care and Use of Laboratory Animals and received the approval from the Ethics Committee of our hospital (DWLL-2023-084).

### 2.16 Immuohistochemical staining

Paraffin sections of xenotumor tissue were dewaxed and rehydrated in xylene and fractional alcohol. Sections were microwave heated in citric acid buffer and incubated with 3 % H_2_O_2_ at room temperature for 30min. The slices were then incubated with primary antibodies (anti-SRSF7: 1:1000, ab137247, Abcam; anti-Ki67: 1:1000, ab279653, Abcam) overnight. The next day, the slices were incubated with biotin-labeled secondary antibodies. Sections were washed with PBS for 3 times and incubated with DAB and stained with hematoxylin.

### 2.17 Statistics analysis

The experimental data were analyzed using GraphPad Prism 7.0 software. Measurement data were represented by mean ± SD, and independent sample *t*-test was used for the comparison between the two groups. All experiments were independent and repeated at least three times. P less than 0.05 indicated a statistically significant difference.

## 3 Results

Increased expression of circTLK1 and reduced expression of miR-876-3p were discovered in NSCLC cells

**Fig. 1:**
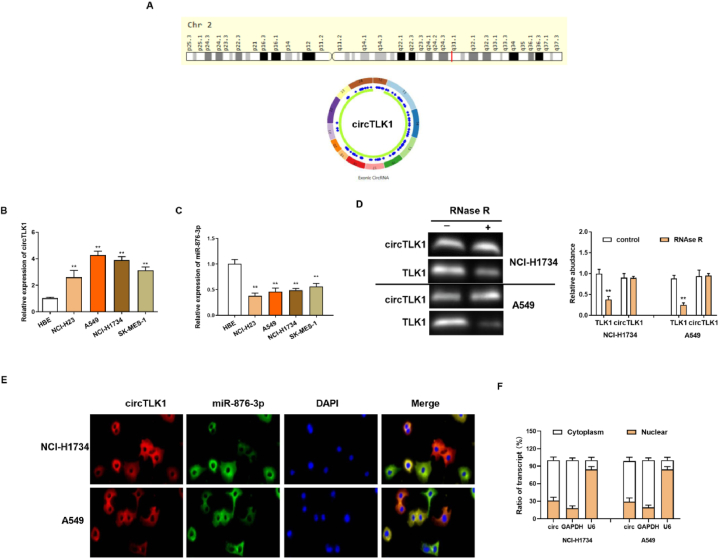
Increased expression of circTLK1 and reduced expression of miR-876-3p were discovered in NSCLC cells (A) Schematic illustrating of the generation of circTLK1. (B, C) RT-qPCR assay for the relative expression of circTLK1and miR-876-3p in NSCLC cells (NCI–H23, A549, NCI–H1734, SK-MES-1) and HBE cells. (D) The expression of circTLK1 and TLK1 was measured by a RT-qPCR assay upon RNase R treatment (The original image is provided in the Supplementary file S-Fig. 1D). (E) The cellular distribution of circTLK1 and miR-876-3p was analyzed by FISH. (F) The subcellular location of circTLK1 was examined by RT-qPCR. **p < 0.01.

Hsa_circTLK1 is derived from TLK1 mRNA, located on chromosome 2: 171879381-171902872 (Fig. 1A). Compared with normal cells, circTLK1 was upregulated (Fig. 1B) and miR-876-3p was downregulated in four NSCLC cells lines (A549, SK-MES-1, NCI–H1734, NCI–H23 cells) (Fig. 1C). To verify the characteristics of circTLK1, the total RNA was digested by RNase R and then detected by RT-qPCR. It was found that the abundance of linear RNA was visibly decreased, while that of circTLK1 was basically unchanged (Fig. 1D). Furthermore, Cytoplasmic localization showed that circTLK1 was mainly located in the cytoplasm (Fig. 1E and F). Our results indicated that circTLK1 was upregulated and miR-876-3p was downregulated in NSCLC cells, suggesting that abnormally expressed circTLK1 and miR-876-3p may be involved in the progression of NSCLC.

### 3.1 MiR-876-3p was targeted by circTLK1

**Fig. 2:**
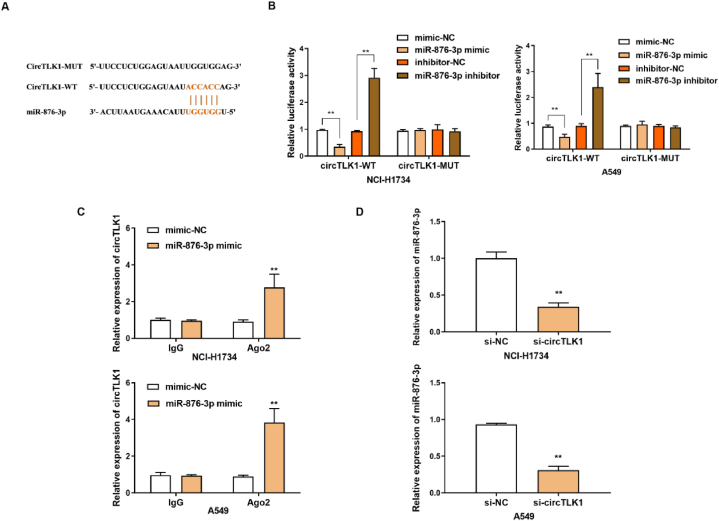
MiR-876-3p was targeted by circTLK1 (A) The binding sites between miR-876-3p and circTLK1 were predicted by Circular RNA Interactome. (B) Dual-luciferase reporter assay for the luciferase activity of WT-circTLK1 and MUT-circTLK1. (C) CircTLK1 enrichment was detected by RNA immunoprecipitation assay in cells transfected with miR-NC or miR-876-3p. (D) The expression of miR-876-3p was measured by RT-qPCR. **p < 0.01.

As shown in Fig. 2A, the binding sites were predicted by Circular RNA Interactome (https://circinteractome.nia.nih.gov/index.html). Dual luciferase reporter gene assay displayed that miR-876-3p mimic notably decreased the luciferase activity of NCI–H1734 and A549 cells transfected with WT-circTLK1 but not MUT-circTLK1. While, miR-876-3p inhibitor increased the luciferase activity of WT-circTLK1, and had little effect on MUT-circTLK1 (Fig. 2B). To further confirm this interaction, anti-AgO2 RIP was also performed on NCI–H1734 and A549 cells transfected with miR-876-3p, and the presence of circTLK1 was detected by RT-qPCR (Fig. 2C). Furthermore, when circTLK1 was inhibited by interfering RNA, the expression of miR-876-3p in NCI–H1734 and A549 cells was significantly increased (Fig. 2D). Therefore, we educed that miR-876-3p was targeted by circTLK1.

### 3.2 Reduced of circTLK1 hindered the malignant progression of NSCLC cells by regulating miR-876-3p

**Fig. 3:**
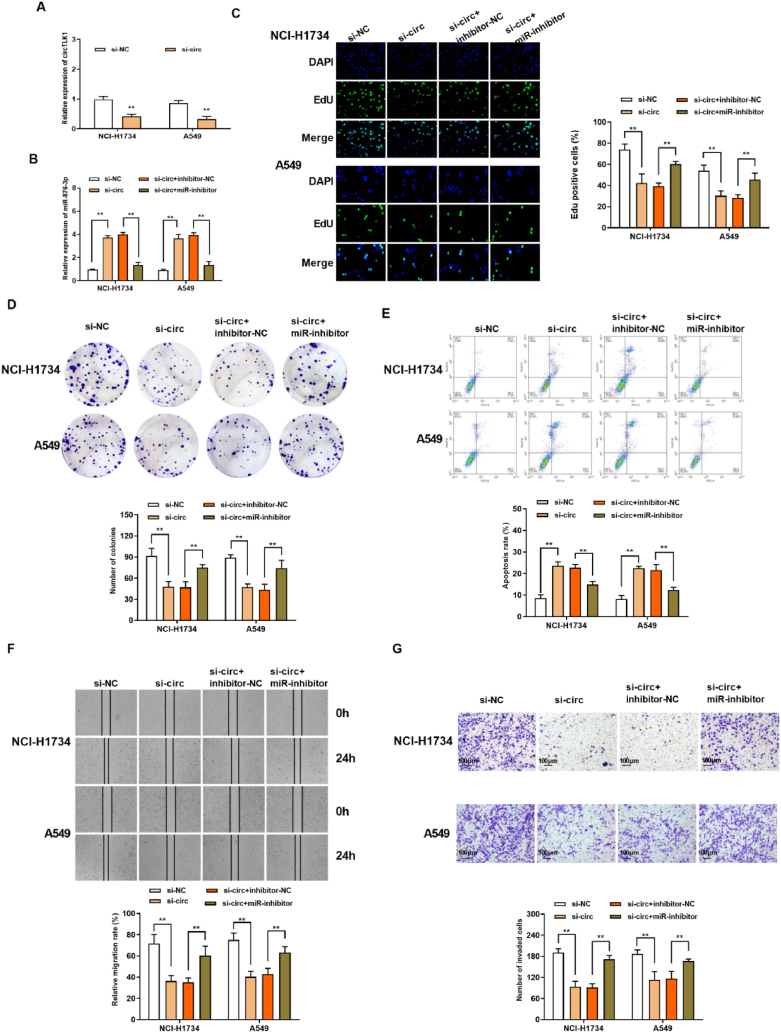
Reduced of circTLK1 hindered the malignant progression of NSCLC cells by regulating miR-876-3p (A) The expression of circTLK1. (B) The expression of miR-876-3p. (C) DNA synthesis was determined by EdU assay. (D) Cell plate clone formation assay for the clonogenicity of transfected cells. (E) The cell apoptosis of NCI–H1734 and A549 cells was evaluated by FCM. (F) The migrated ability of NCI–H1734 and A549 cells was assessed through Wound healing test. (G) The cell invasion of NCI–H1734 and A549 cells was evaluated by Transwell assay. **p < 0.01.

To obtain the evidence of circTLK1 involvement in NSCLC cells, the expression of circTLK1 was successfully knocked down in NCI-H1734 and A549 cells (Fig. 3A). Moreover, circ-TLK1 knockdown reduced the expression level of miR-876-3p, but transfection with miR-inhibitor disrupted the effect of circ-TLK1 knockdown (Fig. 3B). In the following work, we measured the effects of circTLK1 knockdown and co-transfection of miR-876-3p on the proliferation, invasion, migration and apoptosis in NCI–H1734 and A549 cells. The proliferation of lung cancer cells was evaluated by EdU assay. The results displayed that si-circTLK1 decreased the DNA synthesis, and the inhibitory effect was reversed after the addition of miR-876-3p inhibitor (Fig. 3C). Also, colony formation was decreased by si-circTLK1, but reversed after transfection with miR-876-3p inhibitor (Fig. 3D). On the contrary, cell apoptosis was promoted by si-circTLK1, and suppressed by the transfection of miR-876-3p inhibitor (Fig. 3E). In addition, Wound healing test and Transwell assay showed that si-circTLK1 blocked cell migration and invasion, and miR-876-3p inhibitor reversed this change (Fig. 3F and G). Collectively, reduced of circTLK1 hindered the progression in NSCLC cells by regulating miR-876-3p.

### 3.3 SRSF7 was a target of miR-876-3p

**Fig. 4:**
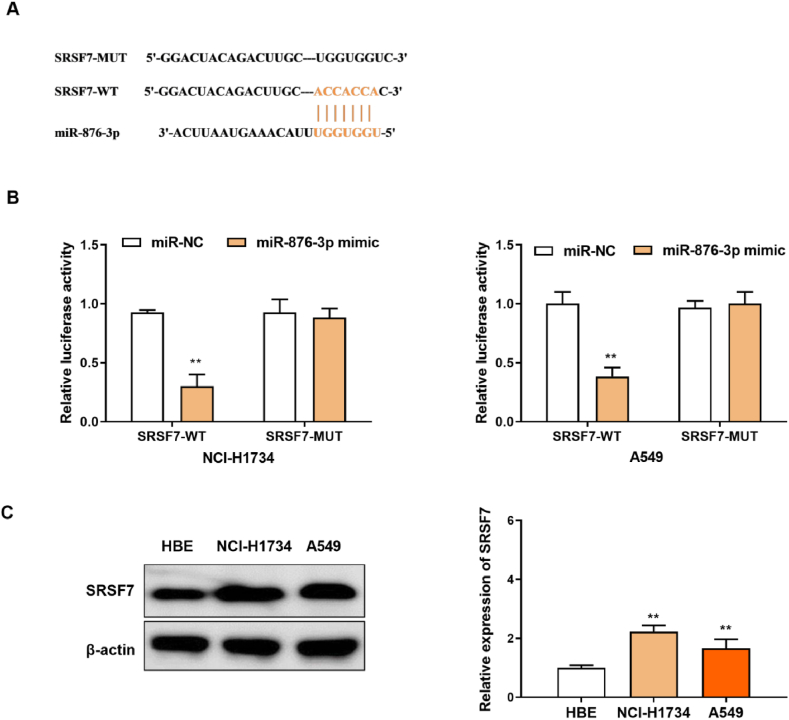
SRSF7 was a target of miR-876-3p (A) The potential binding sites between SRSF7 3’UTR and miR-876-3p were predicted by TargetScan. (B) Dual-luciferase reporter assay for the luciferase activity of WT- SRSF7 and MUT-SRSF7. (C) Western blotting assay for the protein expression of SRSF7 in NCI–H1734 and A549 cells (The original image is provided in the Supplementary file S-Fig. 4C). **p < 0.01.

TargetScan showed that there were binding sites between the 5’ UTR of miR-876-3p and the 3’ UTR of SRSF7-WT (Fig. 4A). To validate the relationship, a dual-luciferase reporter gene assay was performed. According to the results, the luciferase activity of SRSF7-WT was repressed by miR-876-3p mimic, while the luciferase activity of SRSF7-MUT did not changed (Fig. 4B). Moreover, the expression of SRSF7 was higher in NCI–H1734 and A549 cells than in HBE cells (Fig. 4C). Hence, SRSF7 was a target of miR-876-3p.

### 3.4 SRSF7 silencing suppressed the deterioration of NSCLC cells

**Fig. 5:**
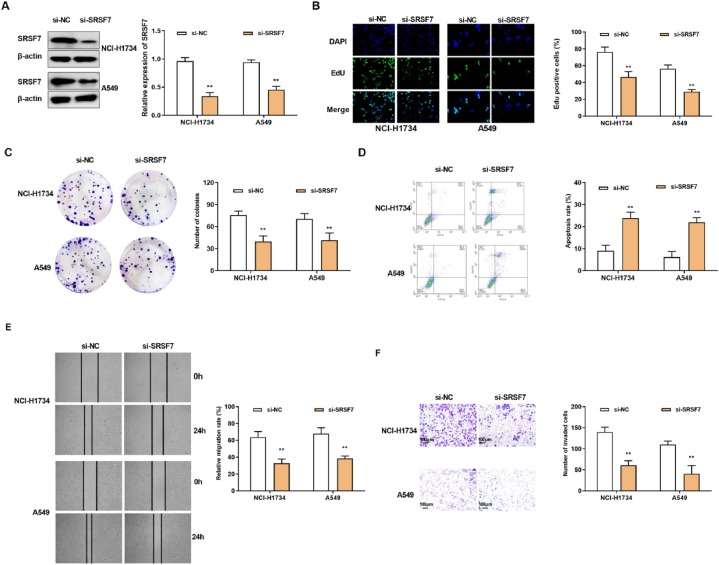
SRSF7 silencing suppressed the deterioration of NSCLC cells (A) The protein expression of SRSF7 (The original image is provided in the Supplementary file S-Fig. 5A). (B) EdU assay. (C) The effect of si-SRSF7 on the clonogenicity was evaluated by Cell plate clone formation assay. (D) The effect of si-SRSF7 on cell apoptosis was evaluated by FCM. (E) The effect of si-SRSF7 on cell migration ability was assessed through Wound healing test. (F) The effect of si-SRSF7 on cell invasion was evaluated by Transwell assay. **p < 0.01.

The expression of SRSF7 was knock downed in NCI–H1734 and A549 cells (Fig. 5A). EdU and cell plate clone formation assay showed that knockdown of SRSF7 distinctively restrained cell proliferation (Fig. 5B and C). SRSF7 silencing promoted the apoptosis of NCI–H1734 and A549 cells (Fig. 5D). Besides, cell invasion and migration were restrained in NCI–H1734 and A549 cells transfected with si-SRSF7 (Fig. 5E and F).

### 3.5 CircTLK1 regulated the oncogenic activities via the miR-876-3p/SRSF7 axis in NSCLC

**Fig. 6:**
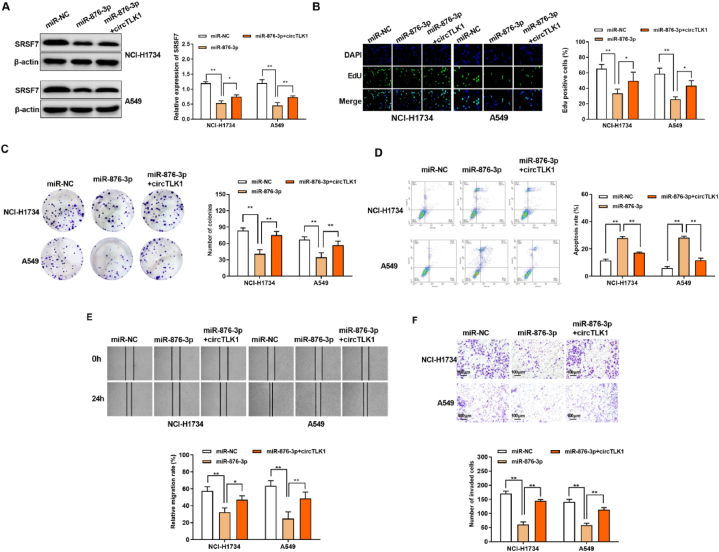
CircTLK1 regulated the oncogenic activities via the miR-876-3p/SRSF7 axis(A) Western blotting assay for the protein expression of SRSF7 in NCI–H1734 and A549 cells (The original image is provided in the Supplementary file S-Fig. 6A). (B) EdU assay. (C) Cell plate clone formation assay for the clonogenicity of transfected cells. (D) The cell apoptosis of NCI–H1734 and A549 cells was evaluated by FCM. (E) The migrated ability of NCI–H1734 and A549 cells was assessed through Wound healing test. (F) The cell invasion of NCI–H1734 and A549 cells was evaluated by Transwell assay. **p < 0.01.

To test the role of circTLK1/miR-876-3p/SRSF7 in the progression of NSCLC, circTLK1 and miR-876-3p were co-transfected into NCI–H1734 and A549 cells. As presented in Fig. 6A, miR-876-3p suppressed the expression of SRSF7, and SRSF7 expression was increased after co-transfection with circTLK1. According to functional test results, miR-876-3p reduced the proliferation (Fig. 6B and C), migration (Fig. 6E) and invasion (Fig. 6F) in NCI–H1734 and A549 cells but promoted apoptosis rate (Fig. 6D). Compared with the effect of miR-876-3p, co-transfection of circTLK1 showed the opposite effects on NSCLC cell proliferation, motility and apoptosis (Fig. 6B–F). The results indicated that circTLK1 regulated the oncogenic activities via the miR-876-3p/SRSF7 axis in NSCLC.

### 3.6 CircTLK1 activated T cells in NSCLC cells

**Fig. 7:**
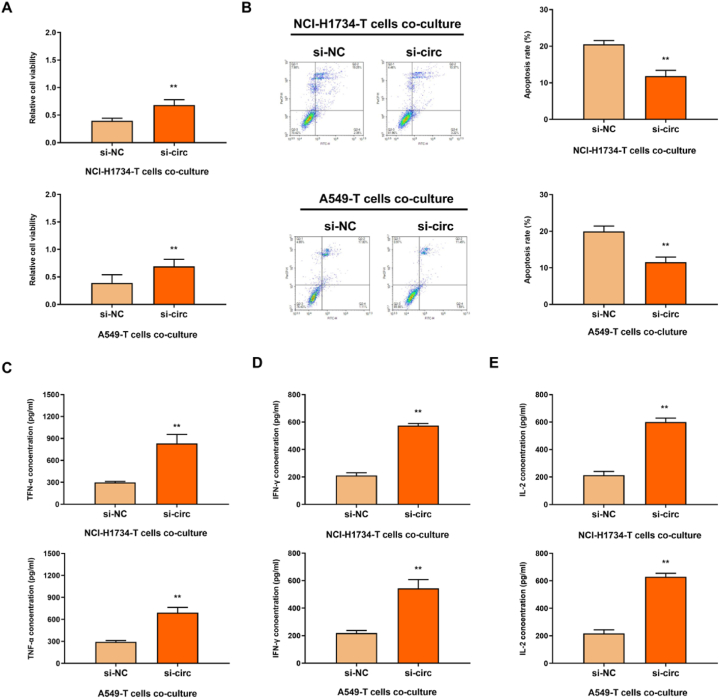
CircTLK1 activated CD8^+^ T cells in NSCLC cells (A) CCK-8 assay. (B) FCM assay. (C–E) The expression levels of IFN-γ, IL-2 and TNF-α were detected by ELISA. **p < 0.01.

To investigate the role and mechanism of circTLK1 in immune escape of NSCLC, si-circTLK1 was transfected into A549 and NCI–H1734 cells co-cultured with T cells. The results of CCK-8 showed that, the proliferation activity of T cells in si-circ group was remarkably higher than that in si-NC group (Fig. 7A). And simultaneously, the apoptosis rate of T cells in si-circ group was observably lower than that in si-NC group (Fig. 7B). IFN-γ, IL-2 and TNF-αlevels of NSCLC cells and co-cultured T cells were determined by ELISA. The levels of IFN-γ, IL-2 and TNF-α in the co-cultured cells transfected with si-circTLK1 was distinctly higher than the si-NC group (Fig. 7C–E). These results indicated that circTLK1 silencing could improve the activity of T cells and induce their secretion of inflammation-related factors, thus affecting the immune escape of NSCLC cells.

### 3.7 CircTKL1 knockdown suppressed the xenograft growth *in vivo*

**Fig. 8:**
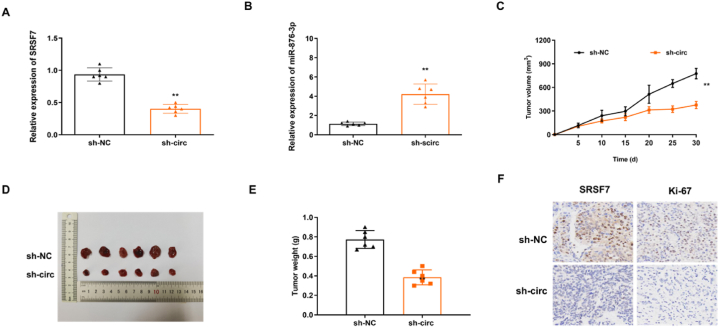
CircTKL1 knockdown suppressed the xenograft growth *in vivo* (A) The expression of circTLK1 in xenograft tumors was detected by RT-qPCR. (B) The expression of miR-876-3p in xenograft tumors was detected by RT-qPCR. (C) Tumor volume of nude mice was recorded every 5 days. (D, E) Tumor weight was measured after injection for 30 days. (F) IHC analysis was used to detect the expression of SRSF7 and Ki67 in tumor tissues. **p < 0.01.

The effect of circTLK1 on tumorigenesis *in vivo* was determined by tumor transplantation in nude mice. NCI–H1734 cells transfected with sh-circTLK1 or sh-NC were injected into nude mice. The results showed that sh-circTLK1 was reduced in tumor tissues (Fig. 8A), and sh-circTLK1 increased the expression of miR-876-3p (Fig. 8B). Then, the size and volume of tumor growth were measured. The results showed that the tumors in the sh-circTLK1 group were smaller than that in the sh-NC group (Fig. 8C–E). As shown in IHC images, the expression levels of SRSF7 and Ki-67 were decreased in the circTLK1 knockdown group (Fig. 8F). Hence, circTKL1 knockdown was found to suppress the xenograft growth *in vivo*.

## 4 Discussion

CircRNA has become a research hotspot in the field of RNA, and has attracted more and more attention due to its specificity of expression, complexity of regulation and important role in disease occurrence. However, the underlying mechanisms of circRNAs' biological function in NSCLC remain elusive. In this study, we found that circTLK1 (derived from the oncogene TLK1) plays an important role in NSCLC, and its stability was confirmed by Rnase R digestion.

CircTLK1 is a newly discovered circRNA that is involved in the development and progression of various tumors. For instance, circTLK1 enhances renal cell carcinoma cell proliferation and metastasis via the regulation of miR-495-3p and CBL [17]. CircTLK1 mediates PBX2 activation of JAK/STAT signaling to promote glioma progression [18]. However, the mechanism of circTLK1 in NSCLC has not been reported. To investigate the mechanism of circTLK1, we downregulated circTLK1 expression in NCI–H1734 and A549 cells. Knockdown of circTLK1 played an inhibitory role in cell proliferation, invasion and migration, but accelerated cell apoptosis in NCI–H1734 and A549 cells. Furthermore, circTLK1 knockdown prohibited xenograft formation *in vivo*. Therefore, it is speculated that circTLK1 may be an oncogene in the development of NSCLC.

CircRNA can target miRNA and negatively regulate its expression, which together affect the development of tumors. In this study, miR-876-3p was confirmed to be the target miRNA of circTLK1. Studies have shown that miR-876-3p has specific effects in a variety of cancers. For example, miR-876-3p blocks the malignant activity of colorectal cancer cells, and is closely related to TNM staging, lymph node metastasis, and perineuronal infiltration [15]. Overexpression of miR-876-3p restraines the growth of pancreatic cancer cells and increases apoptosis by regulating JAG2 [19]. In NSCLC cells, miR-876-3p is uncovered to be downregulated, and reverses the promoting effect of circ_0016760 [20]. Our results demonstrated that miR-876-3p suppressed cell proliferation, migration and invasion, but promoted cell apoptosis. This was consistent with previous findings that miR-876-3p plays an inhibitory role in NSCLC [21,22]. In terms of mechanism, circTLK1 level was directly interacted with miR-876-3p.

Next, the downstream target gene of miR-876-3p was explored. Bioinformatics software predicted that there was a binding site of miR-876-3p in the 3'-UTR region of Serine/arginine-rich splicing factor 7 (SRSF7), and the binding was further verified by dual-luciferase reporter gene assay. SRSF7, formerly known as 9G8, is a member of the SR protein family [23]. SRSF7 can shuttle continuously between the nucleus and cytoplasm, and participate in mRNA transport and translation [24]. Multiple studies have shown that SRSF7 may have an active role in cancer cells. Knockdown of SRSF7 affectes the expression of osteopontin splicing variants and thus suppresses the proliferation of renal carcinoma cells [25]. Saijo et al. found that knockdown of SRSF7 increased the expression of p21, independent of p53 [26]. In small-cell lung cancer, the downregulation of SRSF7 induces a mild decrease in cell proliferation [27]. In this article, we found that knockdown of SRSF7 blocked the proliferation, migration and invasion, but promoted cell apoptosis in NSCLC. Like our findings, overexpression of SRSF7 promotes the tumor promoting effect of MALAT1 on NSCLC cells [28]. However, our study found that the action of SRSF7 was restrained by miR-876-3p in NSCLC.

The regulation of immune function plays an important role in the occurrence and development of tumor. The immune escape of tumor cells is closely related to T cells, which regulate the body's immune response [29]. IFN-γ, IL-2 and TNF-α are important cytokines that regulate immune response and play an important role in the body's immune response, which secreted by T cells activation [[30], [31], [32]]. It was found that the levels of IFN-γ, IL-2 and TNF-α increased significantly after transfection of si-circTLK1 in co-cultured cells. These results indicated that circTLK1 knockdown could promote T cells activation to secrete cytokines, and inhibit immune escape of tumor cells.

However, the expression levels of circTLK1, miR-876-3p and SRSF7 in NSCLC patients and their correlation still need to be further verified. In addition, *in vivo* experiments are needed to further verify the molecular mechanism of circTLK1 mediating NSCLC immune escape, which will be supplemented in future studies.

## 5 Conclusion

In conclusion, circTLK1 knockdown blocked tumor progression and immune escape, and its mediated mechanism may be related to the regulation of miR-876-3p/SRSF7 axis. This study suggested that circTLK1 was a potential target for the treatment of NSCLC.

## Data availability statement

The data will be made available on request to the corresponding author.

## Funding

None.

## CRediT authorship contribution statement

**Xinzhe Dong:** Data curation, Conceptualization. **Hui Tian:** Investigation, Formal analysis. **Peng Ren:** Project administration, Methodology. **Yanxia Liu:** Software, Project administration. **Lin Wang:** Writing – original draft, Visualization, Software.

## Declaration of competing interest

The authors declare that they have no known competing financial interests or personal relationships that could have appeared to influence the work reported in this paper.
