# Supplementary figures and images for: Downregulation of hsa_circTLK1 represses non-small cell lung cancer progression by regulating miR-876-3p/SRSF7 axis
Source: Heliyon. 2024 May 25;10(11):e31972. doi: 10.1016/j.heliyon.2024.e31972 (PMC11167351; doi:10.1016/j.heliyon.2024.e31972)

**1D-circTLK1-NCI-H1734**


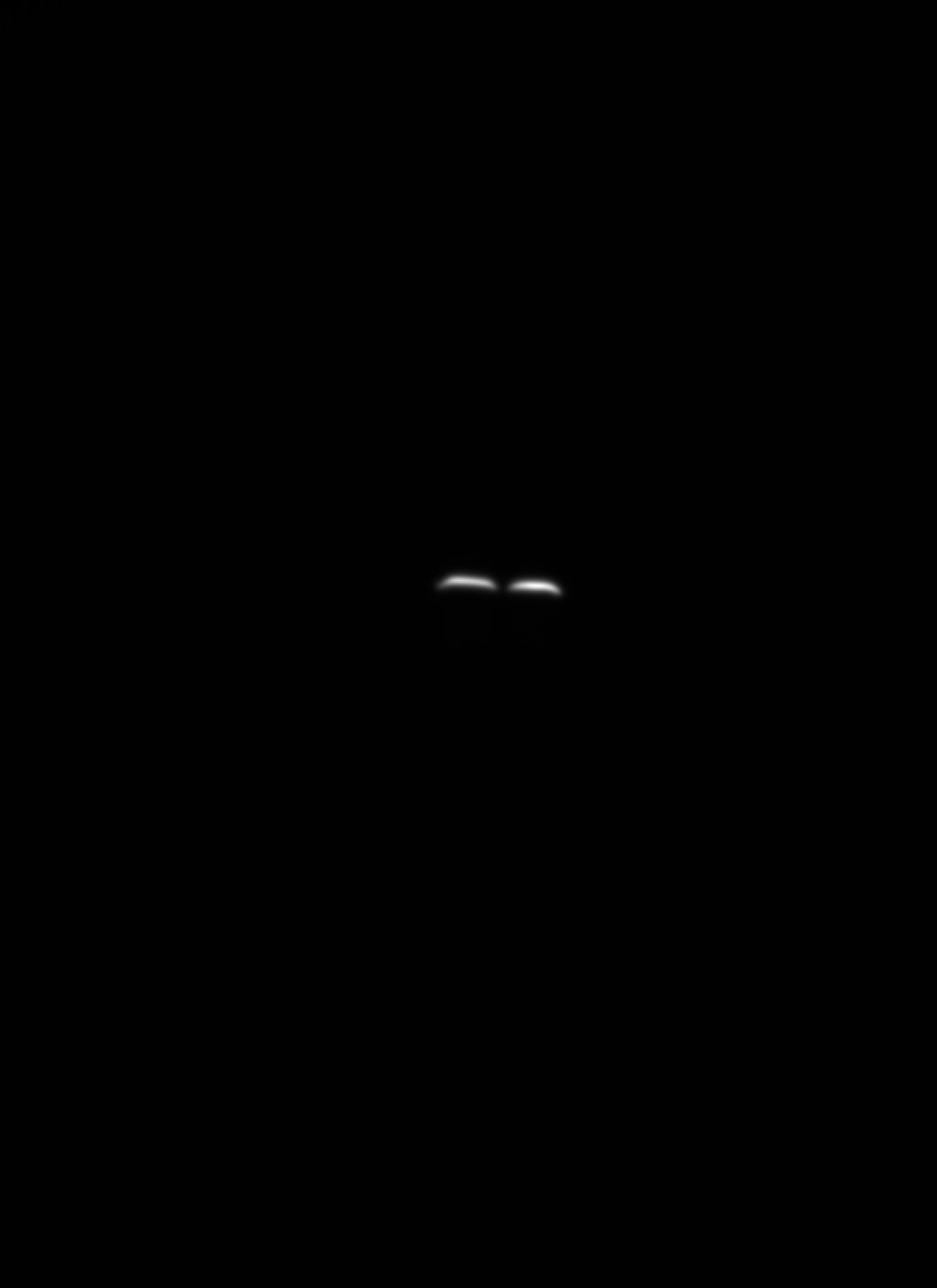


**
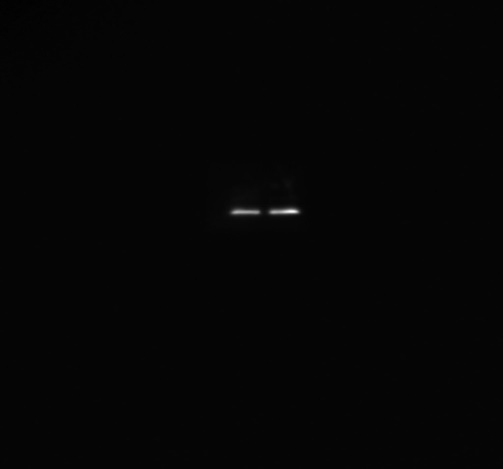
 1D-circTLK1-A549**

**
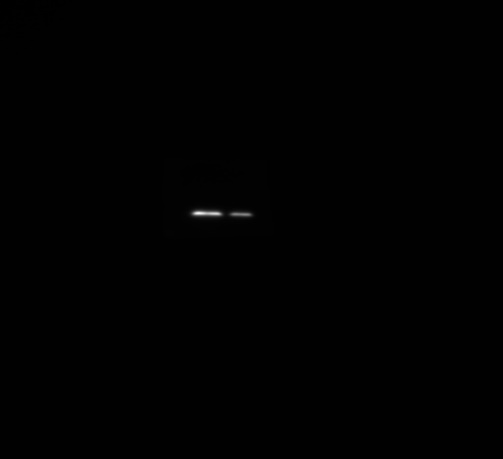
 1D-TLK1-NCI-H-1734**

**
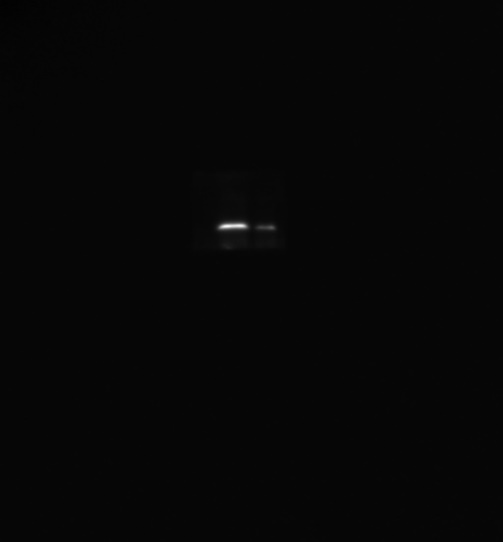
 1D-TLK1-A549**

Supplement: Multimedia component 1 [file mmc1.docx]

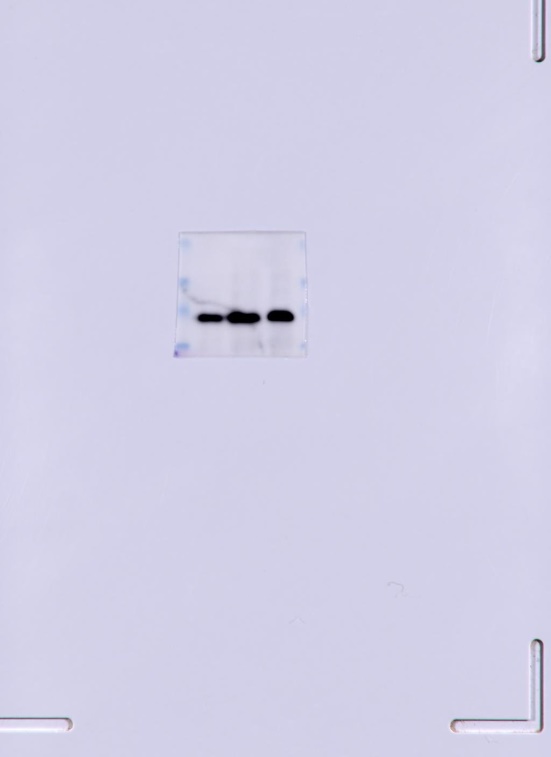
 **4C SRSF7**


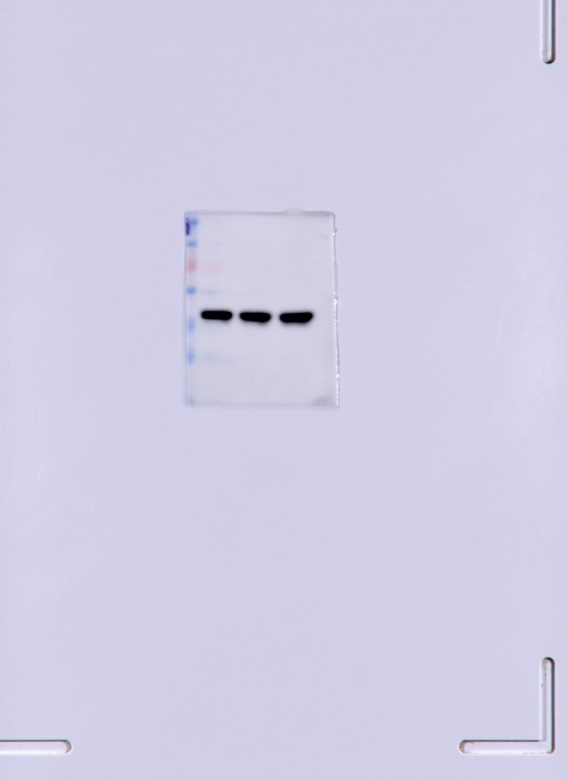
 **4C β-actin**

Supplement: Multimedia component 2 [file mmc2.docx]

**
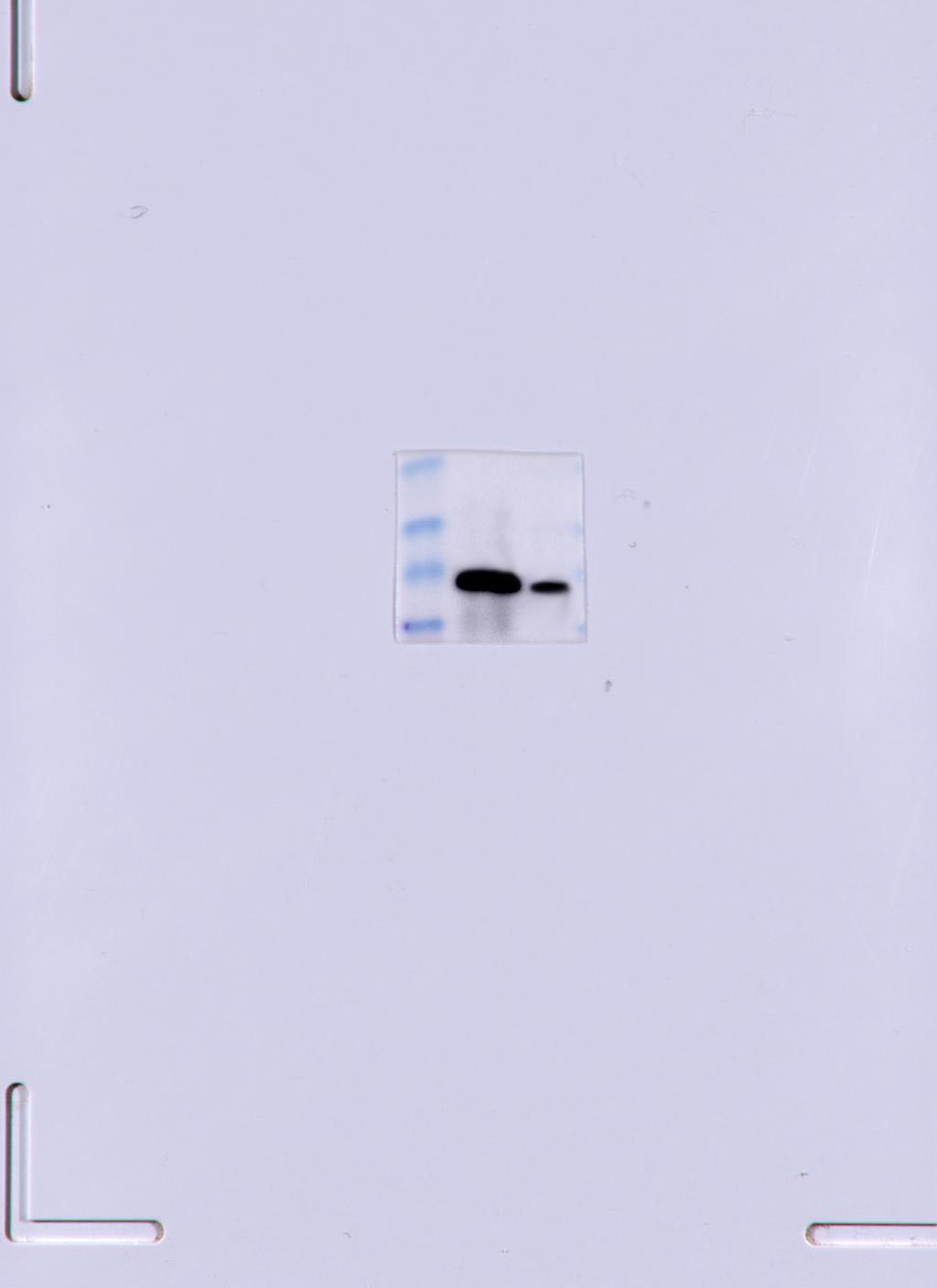
 5A SRSF7 NCI-H1734**

**
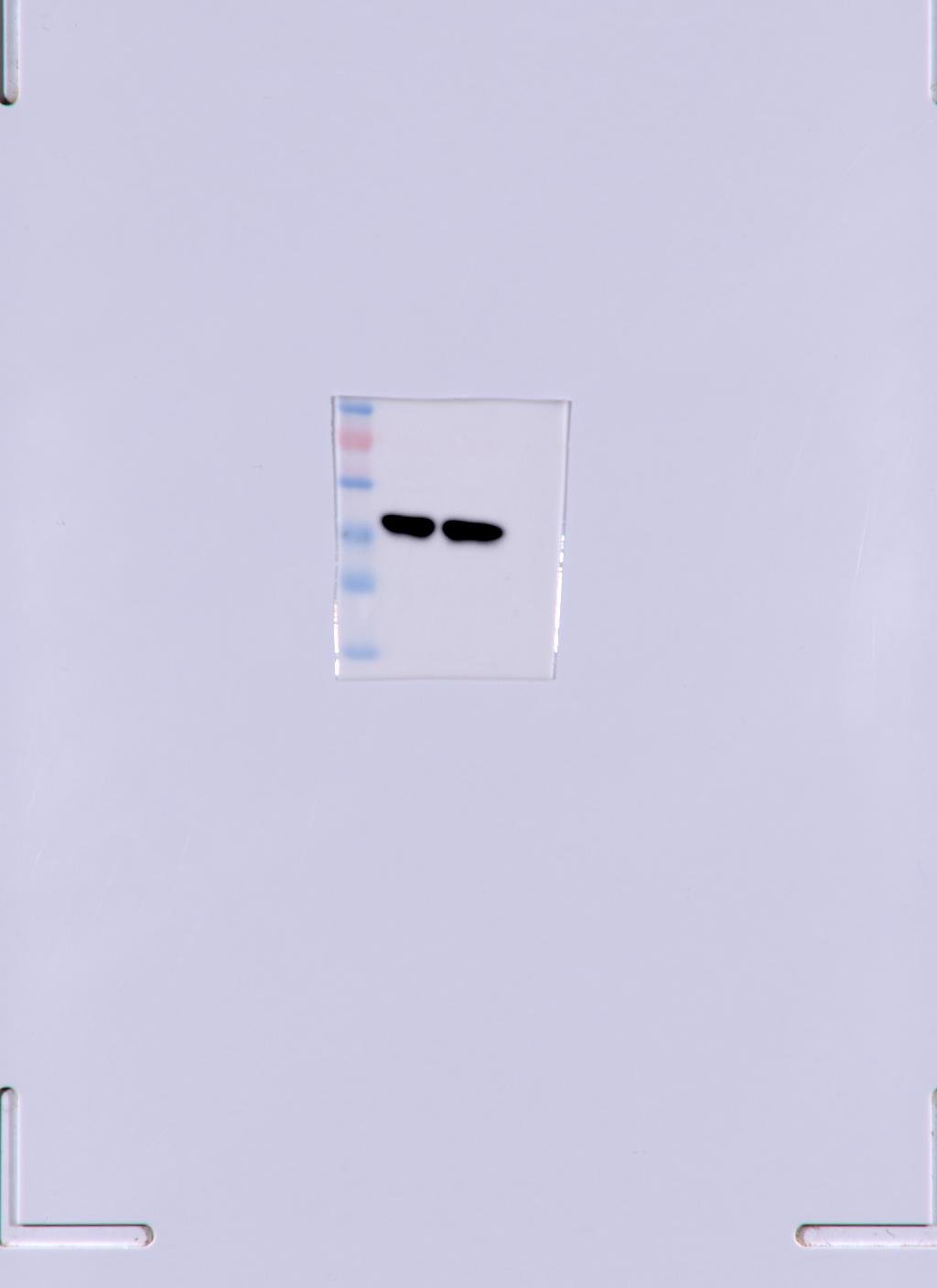
 5A β-actin NCI-H1734**

**
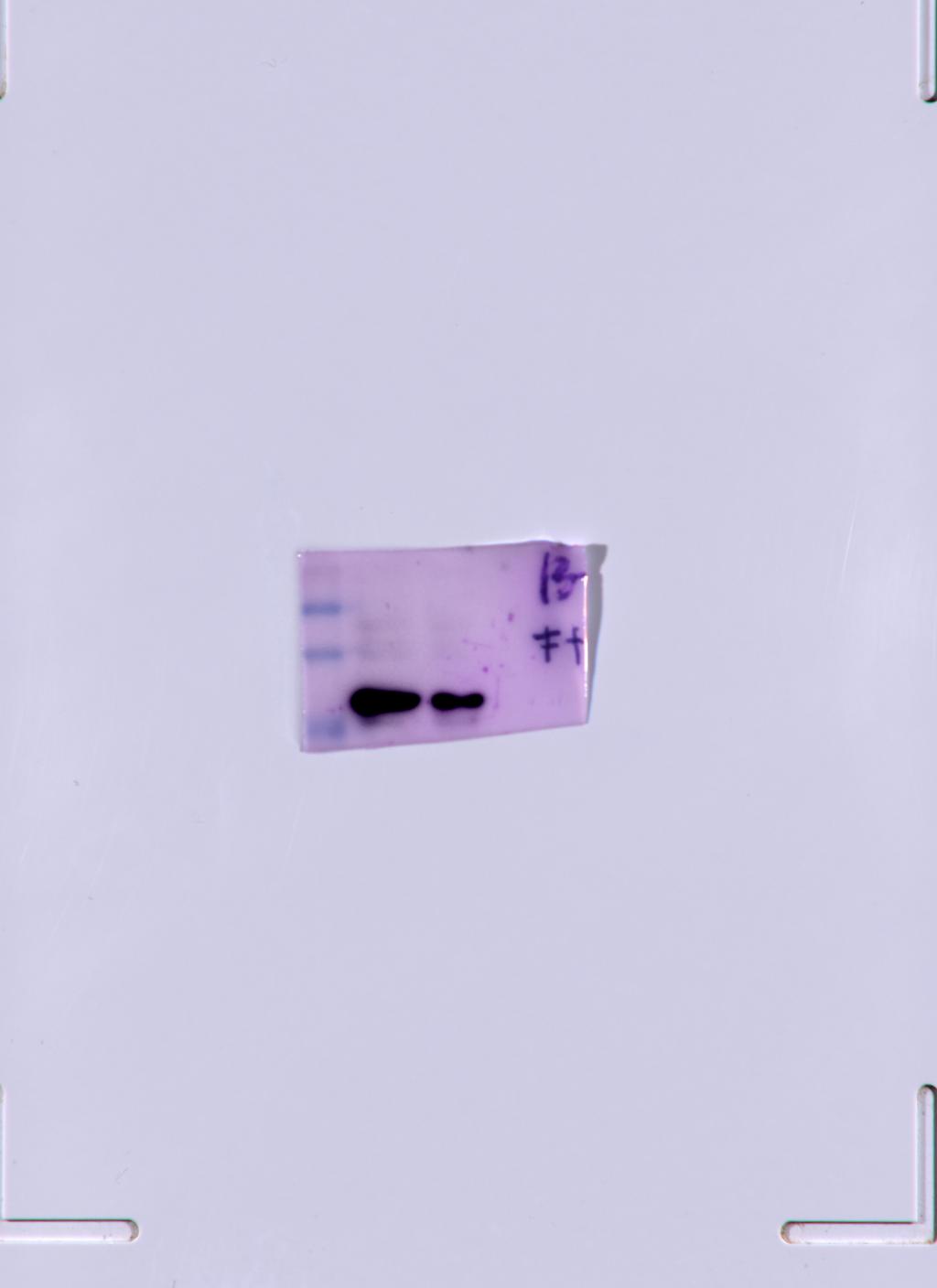
 5A SRSF7 A549**

**
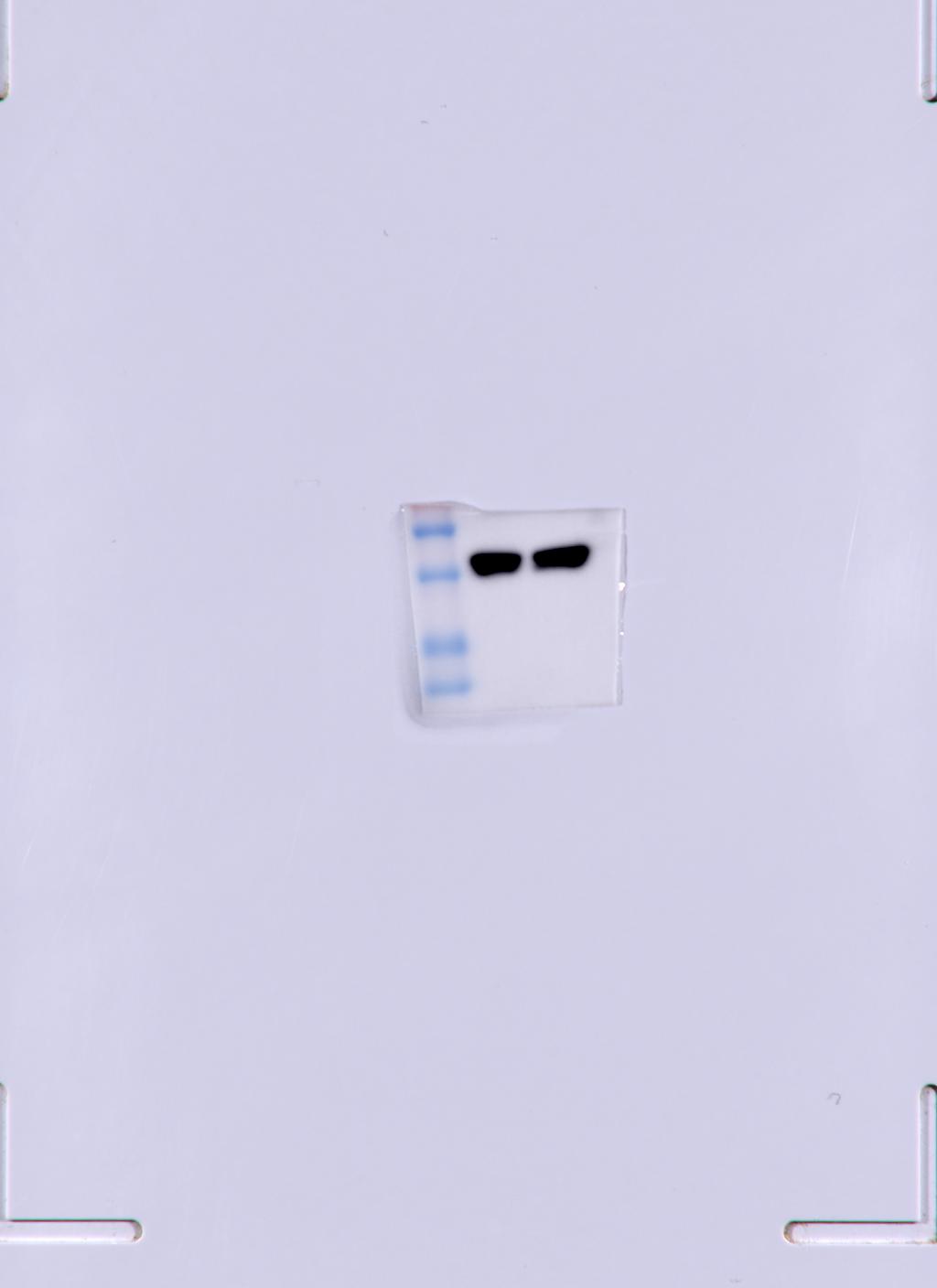
 5A β-actin A549**

Supplement: Multimedia component 3 [file mmc3.docx]

**
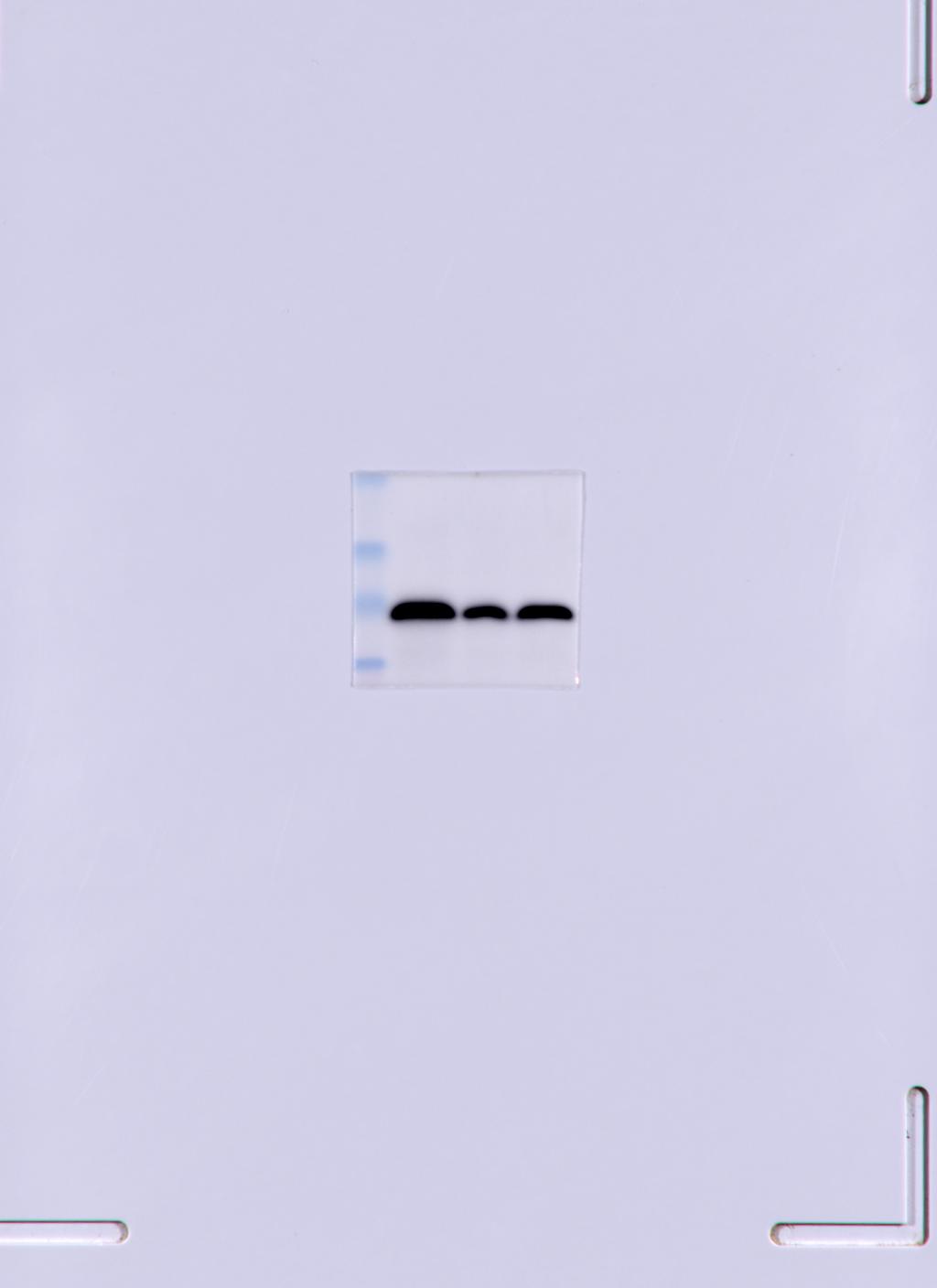
 6A SRSF7 A549**

**
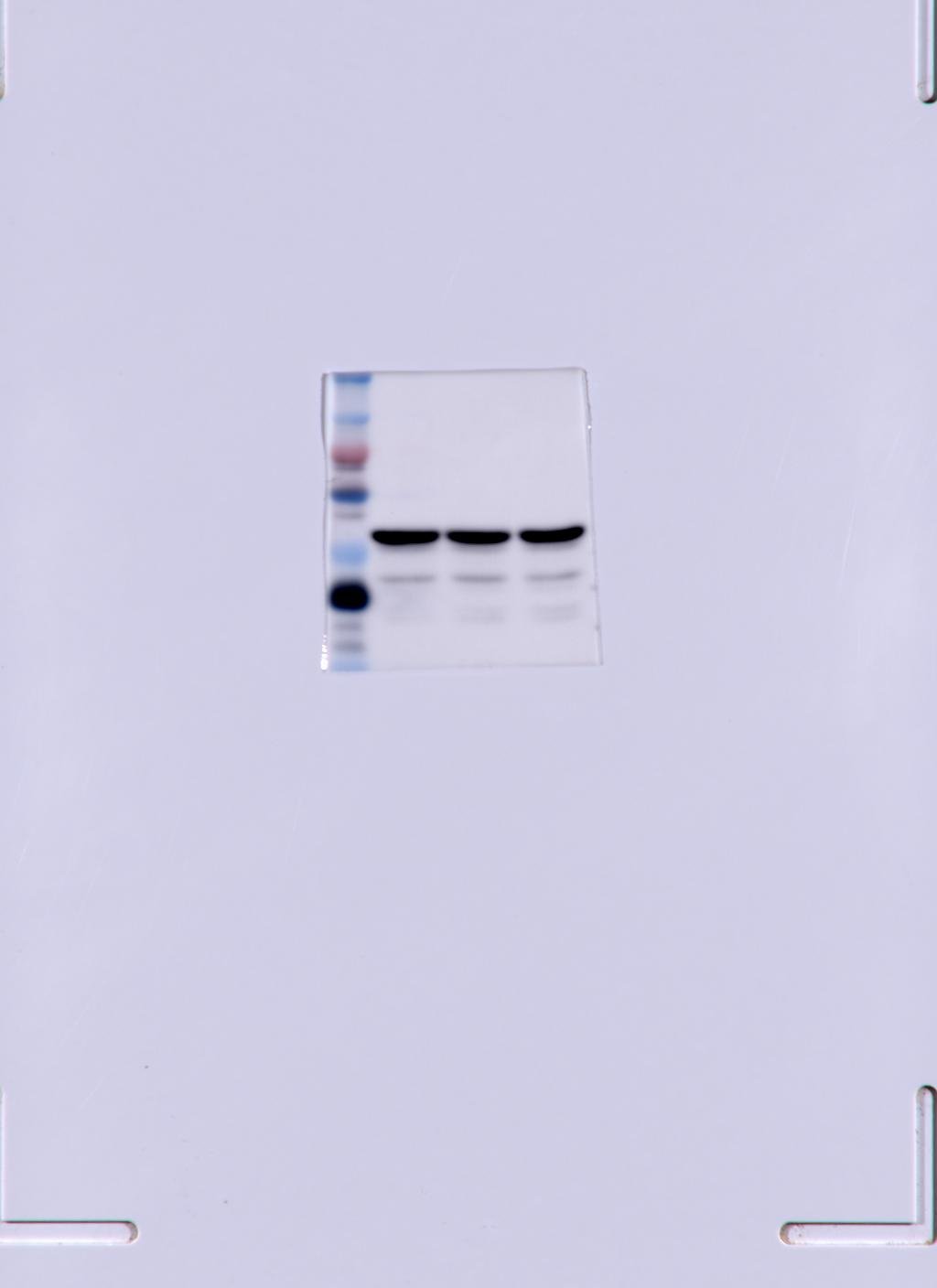
 6A β-actin A549**

**
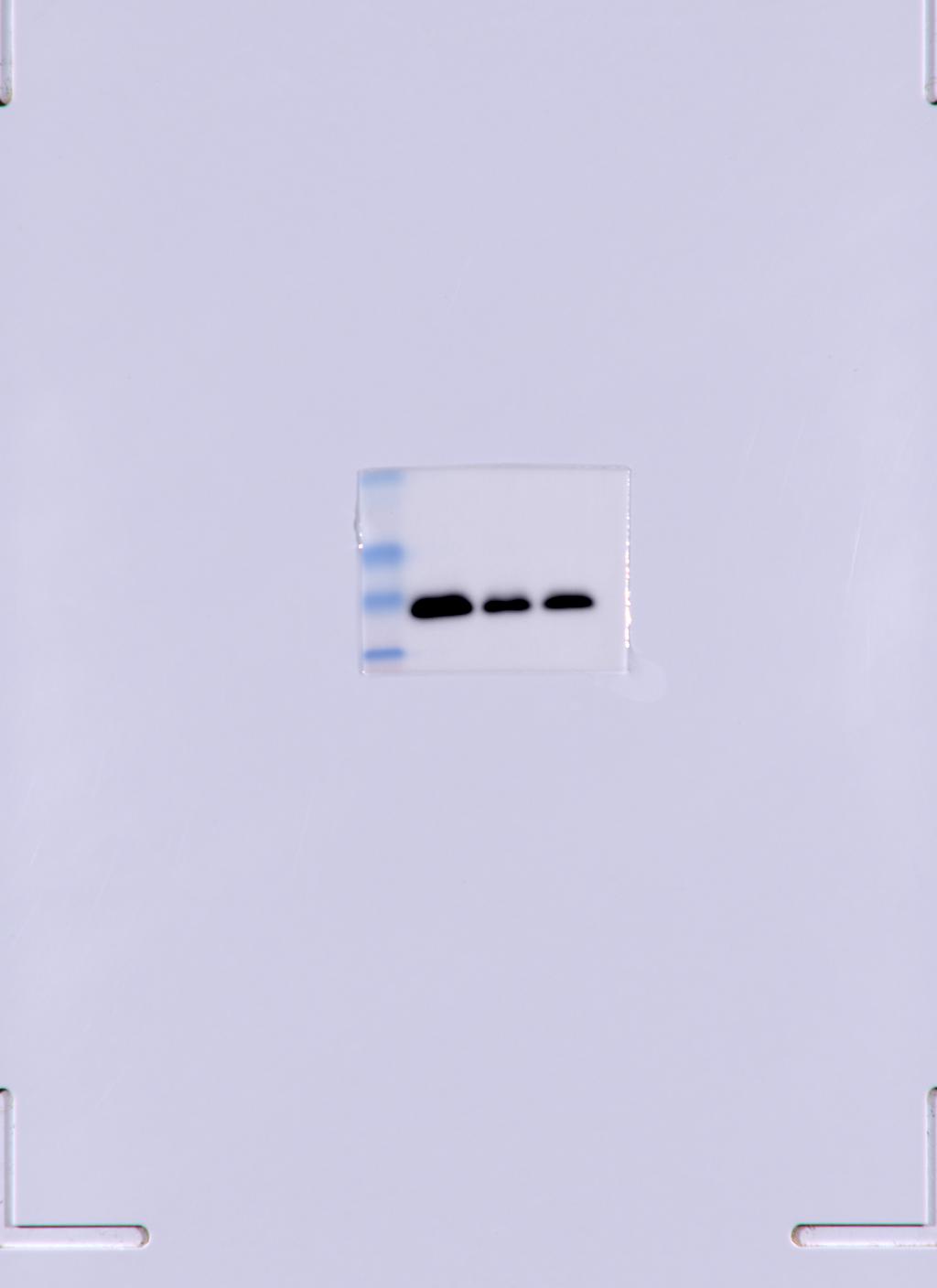
 6A SRSF7 NCI-H1734**

**
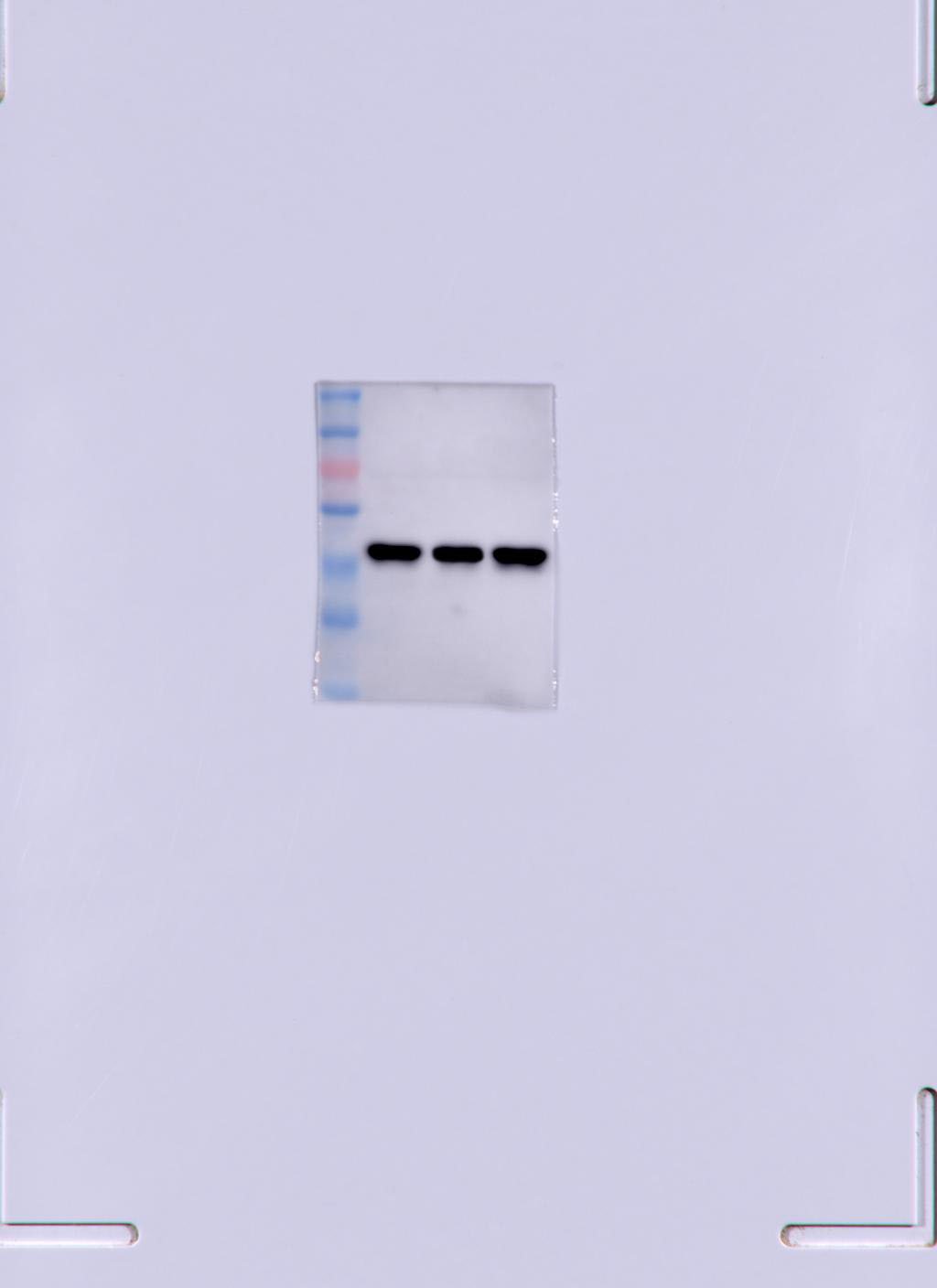
 6A β-actin NCI-H1734**

Supplement: Multimedia component 4 [file mmc4.docx]
